# Rich without Money

**Published:** 1875-06

**Authors:** 


Rich Without Money.—Many a man is rich without money. Thousands of men with nothing in their pockets are rich. A man born with a good sound constitution, a good stomach, a good heart, good limbs, and a pretty good headpiece, is rich. Good bones are better than gold; tough muscles better than silver; and nerves that flash fire and carry energy to every function are better than houses or land. It is better than a landed estate to have the right kind of father or mother. Good breeds and bad breeds exist among men as really as among herds and horses. Education may do much to check bad tendencics or to develop good ones, but it is a greater thing to inherit the right proportion of faculties to start with. The man is rich who has a good disposition—who is naturally kind, patient, cheerful, and hopeful.

---

We see it Stated that white lead paint has been found, after trying almost every plan of treatment hitherto proposed, to be the best and cleanest application for burns. It is to be mixed as for painting, but considerably thicker, and applied with a brush. No doubt it would protect the denuded flesh, as well as it protects wood, but we should decline to advise its employment, as the lead would be likely to be absorbed into the system and do decided injury. There are simple substances enough, without resorting to poisons. A very neat and satisfactory dressing for superficial burns consists in coating the surface with mucilage, and then covering it with powdered lycopodium.

